# How to Achieve Carbon Neutrality in Cities? Evidence from China’s Low-Carbon Cities Development

**DOI:** 10.3390/ijerph20032121

**Published:** 2023-01-24

**Authors:** Huaxing Wang, Chuan Chen, Zhiqiao Xiong, Dandan Li

**Affiliations:** 1 Guanghua Law School, Zhejiang University, Hangzhou 310000, China; 2 Law School, Zhejiang University City College, Hangzhou 310000, China; 3 School of Management, Zhejiang University, Hangzhou 310000, China; 4 School of Economics and Management, Changsha University of Science and Technology, Changsha 410005, China; 5 School of Low-Carbon Economics, Hubei University of Economics, Wuhan 430000, China; 6 Collaborative Innovation Center for Emissions Trading System Co-Constructed by the Province and Ministry, Hubei University of Economics, Wuhan 430000, China

**Keywords:** LLCP, carbon emission, carbon neutral mechanisms, synergistic effect, PSM-DID

## Abstract

Low-carbon city pilots (LCCP) is a key policy for realizing emission peak and carbon neutrality in China, using China’s samples from 280 towns from 2006 to 2016. The article utilizes PSM-DID, mediated effects, and moderating effects approach for validating a CO_2_ reduction effect, mechanisms, and synergistic elements of LCCP. The regression outcomes suggest that (1) LCCP significantly decreases CO_2_ emissions levels and average annual carbon emissions in LCCP fall by 2.6 percent. (2) LCCP focus on reducing carbon emissions by increasing R&D investment, the efficiency of energy, and decreasing the high CO_2_ emissions industry. Among them, the reduction of the high carbon emission industry is mainly FDI, while the reduction of local industry is not obvious. (3) LCCP’s carbon reduction effects suggest a reversed U-shape relationship with city size. Digitalization and marketization of LCCP are crucial to the carbon reduction effect. Carbon reduction and pollution reduction have a strong synergistic effect.

## 1. Introduction

Climate change has increasingly influenced human life. Carbon emission has become a major factor causing global climate warnings. The world’s biggest CO_2_ emitter is currently China [1]. For meeting the challenge of global weather changes, changing the resource-consuming economic development mode, and achieving high-quality development, China’s administration has introduced a range of policy measures for decreasing CO_2_ emissions. LCCP is China’s most important strategy to explore CO_2_ reduction in cities [2].

In terms of the origin of CO_2_ emission, the city is the center of human production and life and the center of high-power expenditure and CO_2_ emission [3]. As China’s urbanization progresses rapidly, cities account for 70–80% of total CO_2_ emissions [4,5] and cities have twice the carbon footprint of rural areas. Therefore, the decarbonization of urban development is critical to reducing CO_2_ emissions in China.

The existing literature mainly studies LCCP from LCCP’s concept [6], LCCP’s development model [7,8], LCCP’s evaluation index [9,10], and LCCP’s effectiveness [11].

Based on the real-life experiences of China’s LCCP, some scholars have conducted many empirical assessments of the effects of LCCP construction in China. Yu and Zhang [12] find that LCCP has a significant active impact on green total factor productivity (TFP) and this effect is greater in a non-resource dependent and coastal city in China. Qiu et al. [13] estimated LCCP’s impact on carbon emission efficiency; LCCP can improve CO_2_ emissions performance by about 1.7%. As the time point of carbon peaking approaches, carbon emission efficiency accelerates. Chen et al. [14] find that LCCP dramatically increased the TFP of locally listed firms.

Although there is a relatively rich existing literature on LCCP, there are still some problems. Firstly, most of the carbon emission data used in existing studies are emissions from China’s cities’ statistical yearbooks; this data includes only CO_2_ emissions from straight power spending, such as air, natural air, electricity, heat, etc. The scope of this data is not comprehensive and may underestimate the extent of CO_2_ emission in cities. Secondly, existing literature does not systematically summarize LCCP’s CO_2_ emissions decline path in theory, but only examines individual potential influencing mechanisms such as innovation and industrial structure. Thirdly, the existing literature does not analyze the synergistic elements of carbon reduction in LCCP; these synergistic factors lead to great differences in the construction of LCCP.

There are three main points of possible innovation in this paper. Firstly, this paper uses CEADs data. This data uses IPCC-recommended methods for computing carbon emissions. The data covers all direct carbon emissions from human socio-economic activities within the administrative boundaries of the city, including 47 socio-economic sectors, 17 kinds of fuel combustion, and cement manufacturing processes [15]. Secondly, this paper theoretically divides the carbon emission reduction paths of LCCP into three types and examines the differences between and within mechanisms. Thirdly, this paper analyzes the synergistic elements of LCCP, which play a key role in the effect of LCCP.

## 2. Policies Setting and Mechanism Assumptions

### 2.1. Policies Setting

Former Chinese President Hu delivered an important speech titled “Working Together to Solve Challenges of Weather Changes”. He said that China would dramatically decrease CO_2_ emissions by 2020 at the 2009 U.N. Climate Summit. Subsequently, China officially announced a 40–45 percent cut in carbon emission intensity in 2020 versus 2005 on 26 November 2019.

**Figure 1:**
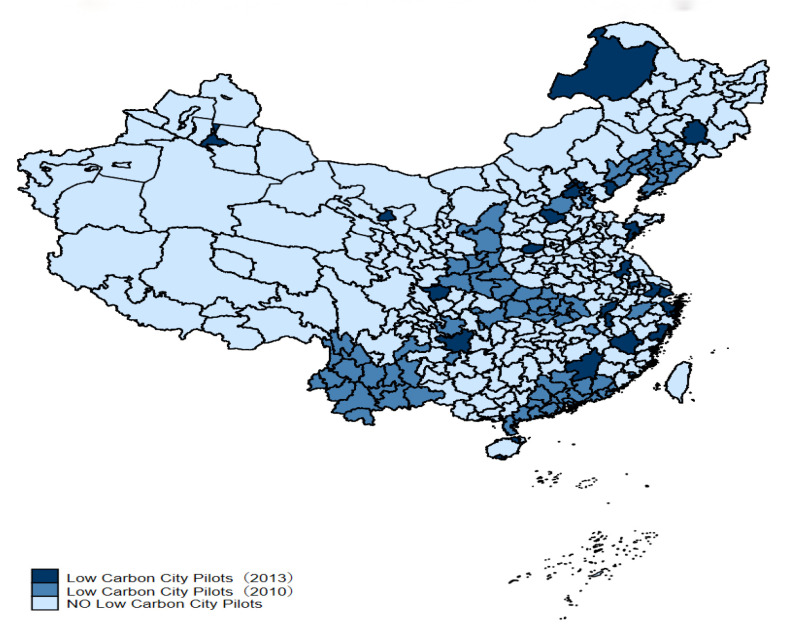
Location distribution of LCCP.

In pursuit of the target to limit carbon emissions, China has introduced a whole set of measures to boost carbon emissions cuts. LCCP is seen as an important policy for China to realize CO_2_ cut targets. NDRC published the first announcements of LCCP in 2010 including eight cities and five provinces, while NDRC published the second announcement of LCCP in 2013 containing thirty-three cities and a province. The particular geographical distribution is presented in Figure 1.

It needs to be explained that China announced the third batch of LCCP in 2017. Due to the current time node, we can only collect data on the cities’ carbon emissions up to 2019; the time frame of the data is too short to fully verify the policy effects of the third batch of LCCP in 2017. So, this paper does not empirically test the policy effect of the third batch of LCCP.

### 2.2. Mechanism Assumptions

#### 2.2.1. Innovative Carbon Reduction

**Figure 2:**
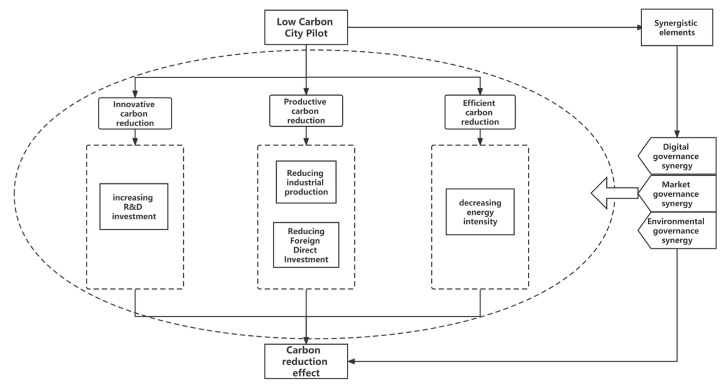
Potential impact mechanisms of LCCP on carbon emission.

As shown in Figure 2, innovative carbon reduction refers to the formation of new technologies, processes, and methods through innovation to replace the original higher carbon emission technologies, achieving lower carbon emission, and zero carbon emission, so far as to negative CO_2_ emissions under the premise of achieving the same output.

The notice issued by NDRC requires LCCP’s government departments to accelerate CO_2_ cut technology innovation [16], boost CO_2_ cut technologies’ R&D, introduction, and widespread application, and positively promote the transformation and upgrading of the conventional industry with the help of CO_2_ cut technology innovation. Meanwhile, government departments ought to track the most recent advancements in CO_2_ cut technology, and positively help industrial enterprises learn advanced foreign technology and make independent innovations.

Existing studies have found that technological progress is an effective means to solve the problem of environmental pollution, especially green technology-oriented innovation [17]. The Porter hypothesis holds that government regulation has a catalytic effect on technological innovation [18]. Bergek et al. [19] find that properly designed government regulations can encourage enterprises to carry out environmentally friendly technological innovation and significantly improve their green technology innovation capabilities. Lee and Min [20] find that green technology innovation can also effectively reduce CO_2_. Hence, we believe that China’s economy and energy system are characterized by high carbonization. The government regulations introduced by the LCCP may promote the advancement of production technology and the upgrading of low-carbon environmental protection technologies, thereby helping to reduce CO_2_.

**Hypothesis** **1:** *LCCP will achieve carbon reduction through technology development.*

#### 2.2.2. Productive Carbon Reduction

As shown in Figure 2, productive carbon reduction refers to the elimination of high resource depletion and high contamination companies, and the decrease of CO_2_ emissions through cutting high resource depletion and high contamination production.

NDCR’s document requires government departments of LCCP to play a comprehensive planning guidance role, adjust the industrial structure, and establish target assessment systems to control CO_2_ emission, as well as implement a goal accountability system to control CO_2_ emission, and allocate emission reduction tasks to the administrative regions under its jurisdiction as well as to key enterprises. Additionally, government departments of LCCP should develop the region’s carbon emission target decomposition and assessment methods and carry out tracking evaluation and emissions abatement assignments completed in time for each assessment responsible body [21].

There are two main theories related to productive carbon reduction. The first is “follow the cost hypothesis”; government regulation intrinsically reduces production efficiency and profits by internalizing the negative externalities of pollution into production costs and affects the industry structure through enterprise entry and exit, production scale adjustment, and resource reallocation [22,23]. The second is the “pollution shelter hypothesis” to evade regulation or reduce the cost of regulation; international differences in environmental standards or the degree of regulation will prompt the transfer of polluting industries across borders, and as a result, it will cause adjustments and changes in the regional industrial structure [24]. So, in this paper, high-energy-consuming production is divided into two parts. One part is from domestic; we use industrial value added to measure. The other part is from abroad; we use FDI to measure.

**Hypothesis** **2:** *LCCP will achieve carbon reduction by reducing high-energy-consuming production.*

#### 2.2.3. Efficient Carbon Reduction

As shown in Figure 2, efficient carbon reduction refers to achieving more outputs with the same amount of carbon emissions or using fewer carbon emissions for the same output by increasing carbon productivity.

NDRC’s document requires government departments of LCCP to improve the resource mix, conserve resources, reduce consumption, enhance efficiency, increase CO_2_ sinks, and incorporate the idea of low-carbon growth into urbanization construction and management, as well as oversee the planning and construction of a public transportation-led model, building energy efficiency design and evaluation technology, and green infrastructure according to low-carbon concepts, and establishing environmental, financial, and tax policies to improve energy efficiency. For example, improving emission standards for all types of enterprises, giving low-interest loans and financial subsidies to enterprises with obvious emission reduction effects, etc.

Existing research shows that the development and utilization of renewable energy and the acceleration of energy transition are important to reduce CO_2_ emissions [25]. Improvements in energy efficiency are a major factor in reducing carbon emissions from China’s transportation sector [26]. Yao et al. [27] find that there are large differences in energy efficiency between provinces in China and improving the energy efficiency of low-energy efficiency provinces will have a greater effect on China’s carbon emission reduction. Akram et al. [28] find that energy efficiency is an important factor in the formation of the environmental Kuznets curve in developing countries.

**Hypothesis** **3:** *LCCP will achieve carbon reduction through energy efficiency.*

#### 2.2.4. Synergistic Carbon Reduction

As shown in Figure 2, synergistic carbon reduction means that LCCP can play a better carbon reduction effect with the deep integration of some factors.

This paper argues that the degree of digitalization, marketization, and ecological construction of cities is closely related to LCCP’s CO_2_ cut effect. The synergy between the degree of digitization and the construction of LCCP is demonstrated by the need for LCCP to improve the management capacity of low-carbon development. It should improve the digital management structure of low-carbon development, establish a work coordination mechanism, prepare the region’s CO_2_ emission list, set up registration, monitoring, and accounting systems for CO_2_ emissions data, and strengthen the digital capacity and talent team building of LCCP. Asongu [29] finds that internet adoption in Africa can open up trade and reduce carbon emissions. Kalmaz and Kirikkaleli [25] emphasize that building “smart cities” using digital technologies is a viable way to achieve carbon reduction.

The synergy between marketization and the construction of LCCP is demonstrated by the need for LCCP to study the use of a market mechanism to facilitate the application of CO_2_ emissions limitation goals. Exploring the systematic mechanism is beneficial to the growth of power saving, emissions control, and low-carbon industries and introducing market-oriented, useful public guidelines, and stimulus policy. Zhang et al. [30] believe that China should establish a unified carbon trading market so that market mechanisms can play a leading role in carbon emission reduction.

The synergistic effect of environmental protection and LCCP construction is demonstrated by the fact that environmental pollutants and CO_2_ emissions have the alike origin and source relationship [31]. LCCP may have synergistic effects of contamination abatement and CO_2_ abatement.

**Hypothesis** **4:** *Digitalization, marketization, and environmental pollution control may have strong synergistic effects with LCCP.*

## 3. Methodology and Data 

### 3.1. Data

This text applies panel data from China’s 280 towns from 2006 until 2016 to assess LCCP’s CO_2_ emissions decrease effects. CO_2_ emissions data in cities originate domain from CEADs [32]. PM2.5 originate from the Chinese Environment Database (CEDS). Other data originate from the “China Energy Statistical Yearbook”, “Urban Statistical Yearbook of China”, “China Statistical Yearbook for Regional Economy”, and “China Industrial Statistical Yearbook”.

### 3.2. Identification Strategy

Since the first and second batches of LCCP were launched at different times, this paper uses a multi-period *DID* approach to estimate LCCP’s influence on CO_2_ emission. Controlling other factors constantly, multi-period *DID* can examine the gap in carbon emissions between experimental regions and non-experimental regions and changes before and after the practice of LCCP. The multi-period *DID* models have been designed as below:

(1) $$Lnco2_{it}=\alpha_{0}+\alpha_{1}DID_{\mathrm{it}}+\sum_{i=1}^{N}b_{j}{Χ}_{it}+\lambda p+\lambda t+\varepsilon_{it}$$

Our text also utilizes the PSM-DID approaches to mitigate the endogenous problems caused by selection bias. The multi-period PSM-DID model is defined as follows:

(2) $$Lnco2_{it}{}^{PSM}=\alpha_{0}+\alpha_{1}DID_{\mathrm{it}}+\sum_{i=1}^{N}b_{j}{Χ}_{it}+\lambda p+\lambda t+\varepsilon_{it}$$

As shown in the above formula, subscripts *i* and *t* indicate region and time, and LnCO_2_ denotes the carbon emissions. *DID* = treatment_i_ × post_it_; they are all dummy variables with a number of 1 or 0, treatment_i_ = 1 means that it is LCCP, and treatment_i_ = 0 means that it is not LCCP. post_it_ = 1 means that it is after the practice of LCCP including the first and second batches, and the rest are defined as 0. *X_it_* is a series of controlled variables. λp denotes regional fixed-effects, λt denotes the time fixed-effects, and $\varepsilon_{\mathrm{it}}^{}$ denotes errors. In addition, to address potential serial correlation and heteroskedasticity, this paper reports on the robust standard error with city clustering; if LCCP significantly reduces local carbon emissions, then ɑ_1_ is significantly negative.

### 3.3. Key Variables and Measurement

Explained variables and explanatory variables. The explained variables are CO_2_ emission and CO_2_ intensity, both taking logarithmic form (lnCO_2_, lncogdp); urban GDP for carbon intensity is the real urban GDP at constant 2006 prices. The explanatory variable is *DID*, which is whether to start LCCP development.

Control variable. Urban carbon emissions are closely related to economic level, economic structure, city size, financial strength, and resource endowment. We need to control these variables to ensure the comparability of carbon emissions between cities. Canadell et al. [33] prove that economic growth has led to a rapid rise in atmospheric CO_2_ concentrations; we use the logarithm of urban GDP (lngdp) and the square of GDP (lngdp2) to weigh the standard of urban economic growth. Cheng et al. [34] find that industrial structure is an important factor affecting CO_2_; this paper utilizes the proportion of secondary industry’s ratio (strind) to weigh the industrial structure of a city. Zhu and Peng [35] find that population has a large impact on CO_2_; we measure the size of the city by the total population’s logarithm (lnpop). Wang et al. [36] find that government finances can affect carbon emissions through economic development and industrial structure; we use fiscal revenue as a percentage of GDP (strpub) to measure the financial strength of a city. Chuai et al. [37] find that land use is a major source of anthropogenic CO_2_; we use administrative area (lnarea) to measure the land resources of a city. The results of critical variables’ descriptive analysis are displayed in Table 1.

In Table 2, by contrasting the carbon emissions between the treatment and control groups and the fourth policy carry out, this paper discovers that the rise in CO_2_ emission, before and after the policy in the treatment groups, is smaller than that of the control groups, indicating that LCCP construction has dampening effects on carbon emissions.

## 4. Empirical Study

### 4.1. Baseline Regression Result

Our article first utilizes *DID* approaches to evaluate the carbon reduction effects of LCCP; benchmark regression result is displayed in Table 3 and all regression results are in control for year fixed-effects and city fixed-effects. In the first column of Table 3, we did not add any control variables, the *DID* coefficient value is −0.029, and it is significant at the level of 5%. In columns (2)–(5) of Table 3, we gradually add control variables, the *DID* coefficients are all negatively significant above the level of 10%, and the regression coefficient is around −0.026, which means that the LCCP may decrease CO_2_ emissions by around 2.6% per year on average. The control variable’s coefficient shows that urban GDP displays a reversed U-type relationship with CO_2_ emission, and both the urban population and industrial structure are positively correlated with CO_2_ emission.

### 4.2. Parallel Trend Test

Drawing on [38], we examine the common trends hypothesis and analyze the dynamic effect of policy based on an event analysis approach. This paper takes the previous year of LCCP practice as a benchmark and constructs the interaction term of the time dummy variable and region dummy variable for 3 years back, the current year, and 3 years forth LCCP practice.

**Figure 3:**
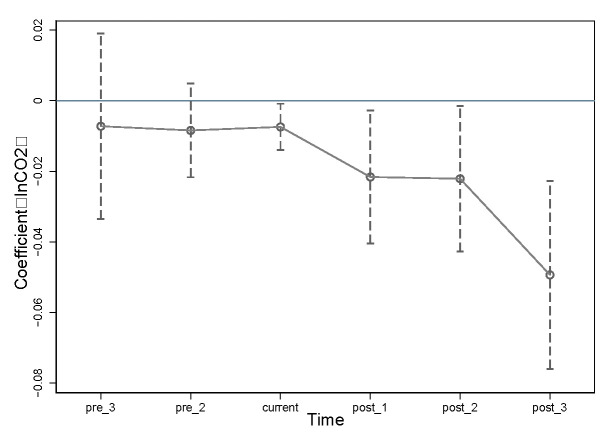
Dynamic effect tests.

As displayed in Table 4 and Figure 3, none of the regression coefficients before the implementation of the LCCP passed significant testing, which indicates that carbon emission changes in the experimental and control group back LCCP practice satisfies the common trends assumption. The coefficients for both the year of LCCP implementation and the three years after implementation are negatively significant above the level of 5%, and in terms of regression coefficients and significance levels, the carbon reduction effect of the third year after LCCP practice has strengthened, meaning that LCCP is increasingly well established.

**Figure 4:**
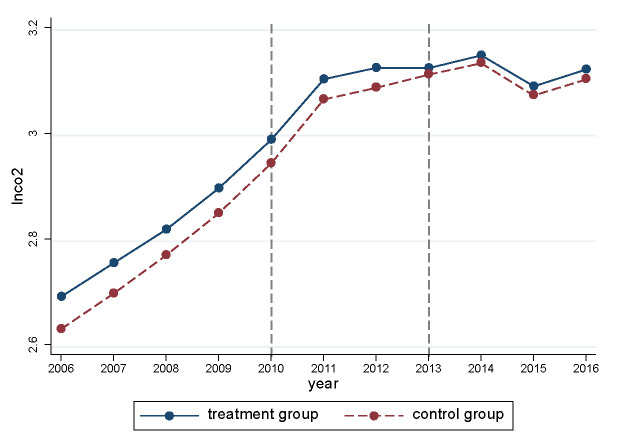
Time series plot.

We also plotted the time trend of the treatment group and the control group in Figure 4. As can be seen in Figure 4, the treatment and control groups maintained a similar growth trend until the policy point in time. After the implementation of the policy, CO_2_ in the treatment group began to decline, compared with those of the control group.

### 4.3. PSM-DID

**Figure 5:**
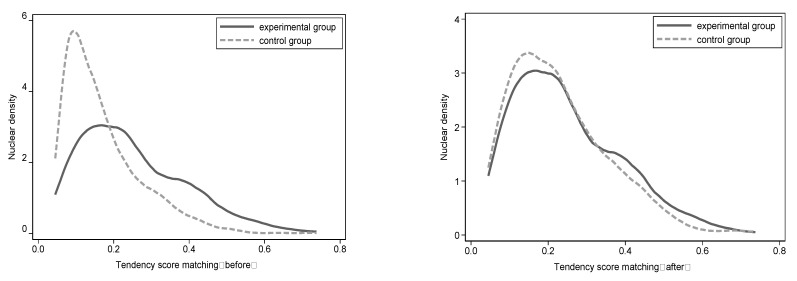
Probability Distribution Density Function of Tendency Score.

We utilize the PSM-DID approach to solve endogenous problems caused by selection bias [39]. We utilize control variables used in the baseline regression as covariates, apply a logit model for propensity score estimation, and perform caliper nearest neighbor matching. The nuclear density curve is shown in Figure 5. We can see that without matching, the Kernel Density curve is not similar between the experimental group and control group, while after matching, the Kernel Density curve is very similar, which indicates the effects of PSM seems well.

Utilizing the post-PSM samples, the outcomes of *DID* regression are displayed in Table 5. We find that the PSM-DID coefficients are negatively significant at the level of 5% and PSM-DID’s coefficient is 0.003 larger than *DID*. It suggests that LCCP’s CO_2_ decrease effects are somewhat underestimated and the basic regression results of this paper are robust.

### 4.4. Placebo Test

To enhance the credibility of benchmark regression outcomes, this paper also needs to perform the placebo test. As shown in Table 6, this paper front-loaded LCCP’s timing for the placebo test and set 2007, 2008, and 2015 as dummy policy years instead of real policy years. The outcomes display that none of the coefficients for fictitious years are significant.

As shown in Table 7, we replace samples for the placebo test and compress the sample time window to examine the stability of the empirical outcomes in this paper. The empirical outcomes indicate that the carbon reduction effect of LCCP is still significant when we compress the sample time window.

### 4.5. Robustness Tests

We utilize substituting the dependent variable approach to conduct a robustness test. CO_2_ intensity means the relative amount of CO_2_ emission per unit of GDP; we can also use the carbon intensity to measure carbon emissions in cities. The outcomes in Table 8 indicate that LCCP’s effect on CO_2_ intensity is also significantly negative.

This paper uses different estimation methods to perform robustness tests. As shown in Table 9, we ran regressions with fixed effects, random effects, maximum likelihood estimation, and population-averaged models. The outcomes suggest each coefficient is negatively significant.

This paper also performs robustness checks by excluding the effects of contemporaneous related policies. The trial of emission rights trading started in 2007, and the regional power conservation and emissions decrease targets under the five-year plans may affect carbon emissions in LCCP. In column 1 of Table 10, this paper regresses only samples in pilot cities of emission rights trading. In column 2 of Table 10, we add the interaction term between the region’s energy reduction target and year as control variables, excluding effects from key control cities’ carbon targets. The outcomes indicate that the effect of LCCP on CO_2_ emission is still negatively significant after excluding the effect of related policies.

## 5. Mechanism Testing

From the above empirical results, we demonstrate that LCCP does have a significant carbon reduction effect. To achieve excellent results in the construction of LCCP, we also need to understand which mechanisms are used in LCCP to achieve carbon emission reductions. This paper utilizes mediation models to examine the mechanism [40]; the mechanism validation model settings are listed below. 

Step 1: Check LCCP’s impacts on carbon emission.

(3) $$Lnco2_{\mathrm{it}}=\beta_{0}+\beta_{1}DID_{it}+\sum_{i=1}^{N}b_{j}X_{\mathrm{it}}+\lambda p+\lambda t+\varepsilon_{\mathrm{it}}$$

Step 2: Check LCCP’s impacts on mechanisms.

(4) $${\mathrm{Mechanisms}}_{\mathrm{it}}=\alpha_{0}+\alpha_{1}DID_{it}+\sum_{i=1}^{N}b_{j}X_{\mathrm{it}}+\lambda p+\lambda t+\varepsilon_{\mathrm{it}}$$

Step 3: Add mechanisms to the baseline regression.

(5) $$Lnco2_{\mathrm{it}}=\gamma_{0}+\gamma_{1}DID_{it}+\gamma_{2}{\mathrm{Mechanisms}}_{\mathrm{it}}+\sum_{i=1}^{N}b_{j}X_{\mathrm{it}}+\lambda p+\lambda t+\varepsilon_{\mathrm{it}}$$

We use R&D expenditure (R&D) to measure innovative carbon reduction [41]. We use energy intensity to measure efficient carbon reduction. We use the logarithm of industrial value added (Lnind) to measure productive carbon reduction from domestic. This paper utilizes the logarithm of FDI (Lnfdi) to evaluate productive carbon reduction from abroad.

The outcomes of mechanisms validation are shown in Table 11. In columns (1), (3), (5), and (7), we can find that the LCCP significantly increases the cities’ R&D investment and significantly decreases energy consumption intensity, foreign direct investment, and industrial value added. In columns (2), (4), (6), and (8), we can find that the coefficient of *DID* becomes insignificant after adding R&D investment, energy consumption intensity, and foreign direct investment to the regression; this shows that LCCP mainly reduces carbon emissions by improving investment in R&D and innovation, energy use efficiency, and decreasing high-emission foreign direct investment. However, the coefficient of *DID* changes less and is still significant, indicating that the reduction in industrial value added of LCCP is not effective in reducing carbon emissions.

## 6. Heterogeneity Analysis

The effect of CO_2_ emissions in cities is closely related to the scale of cities [42]; to test the scale effect of LCCP, we analyze the heterogeneity of carbon emission LCCP’s CO_2_ decrease effects based on city size. We use the city’s resident population as the statistical caliber and divide the city into four categories: Small and medium-sized city, Model II big city, Model I big city, and Super-large city.

On the one hand, small cities may easily achieve economic, industrial, and energy structure promotion and upgrade, leading to a good CO_2_ reduction effect. However, there may be an insufficient scale effect and a higher average cost of carbon reduction. On the other hand, R&D funding in large cities may be more than adequate and the application of green technology innovation may have a larger-scale effect. However, there may face a large conflict between carbon reduction and economic development.

The grouped outcomes in Table 12 indicate that the carbon reduction effects of LCCP present nonlinear relationships with city size. It shows an inverted U-shape relationship; LCCP’s CO_2_ decrease effects rise and then fall as the size of the city increases. The heterogeneity of city size also further validates the results of this paper’s mechanism test, where LCCP is mainly through R&D funding and energy efficiency improvement to achieve carbon emission reduction.

In this paper, we also want to discuss the influence of digitalization and marketization on LCCP construction and the synergy effects of CO_2_ decrease and contamination reduction. We use the percentage of Internet users to evaluate the cities’ digitalization. We utilize IndexMar [43] and the share of SOEs to evaluate the marketization level of cities. This paper used SO_2_ and PM2.5 concentrations to evaluate the cities’ environments. Then, we grouped the samples based on the median; the outcomes are displayed in Table 13 and tested synergistic effects using a moderation-effect model are shown in Table 14. It indicates that high digitization and marketization are conducive to the CO_2_ decrease effects of LCCP. LCCP has a strong synergistic effect on CO_2_ decrease and pollution reduction.

## 7. Conclusions and Policy Implication

LCCP is a practical exploration of China’s green low-carbon growth. Using China’s samples of 280 towns from 2006 to 2016, this paper finds that LCCP can significantly reduce urban CO_2_ emission. The LCCP works through three mechanisms to realize urban carbon reduction including innovating low-carbon production technology, improving energy use efficiency, and restricting investment in the high carbon industry. In restricting investment in the high carbon industry, LCCP plays a strong role in limiting foreign high-energy investments and a weaker role in limiting domestic high-energy investments. We also find that LCCP’s carbon reduction effects display the inverse U-shape relationship with city size. Digitalization and marketization of cities have a strong synergy with LCCP. LCPP also has a significant pollution reduction effect.

The policy recommendations in this article are as follows: (1) LCCP can indeed reduce carbon emissions. As a new type of urban development model, LCCP provides feasible ideas for resolving the tension between urban development, resource conservation, and environmental protection. LCCP should consider both low-carbon production and low-carbon consumption. Building LCCP is key to achieving carbon neutrality. The government departments of many developing countries should formulate green low-carbon development plans, establish a carbon-neutral indicator system, and establish a carbon emission accounting platform to build a low-carbon city. (2) Government departments should be aware that there is some conflict between economic development and LCCP; the low-carbon transition should be achieved on the premise of ensuring GDP growth. Therefore, productive carbon reduction has greater resistance, and innovative carbon reduction and efficient carbon reduction may be a more useful path. The choice of low-carbon transition strategy should be achieved by innovating green technology, reducing the energy intensity (energy saving), and improving the energy mix (by increasing the share of clean energy in the energy consumption structure). (3) LCCP should pay more attention to the promotion of digitalization and marketization and reinforce the synergy effects of carbon reduction and pollution reduction. For Super-large cities, vigorously developing renewable energy and increasing investment in research and development are effective ways to reduce emissions; for big cities, optimizing the industrial structure and improving the quality of urbanization are the keys to reducing carbon emissions; for small and medium-sized cities, to achieve low-carbon development, it is necessary to accelerate the elimination of backward production capacity and accelerate industrial upgrading and transformation based on promoting economic growth.

## Figures and Tables

**Table 1:** Descriptive analysis (Obs: 3058).

Variables	Means	Std	Minimum	Maximum
LnCO_2_	2.96	0.76	0.61	4.86
Lngdp	6.75	0.94	3.95	9.75
lnpop	5.84	0.68	2.86	9.31
strind	0.49	0.11	0.15	0.91
strpub	0.07	0.03	0.02	0.24
Lnarea	9.33	0.82	7.01	12.44

**Table 2:** Comparison of carbon emission between treatment groups and control groups.

	Treatment Groups before the Policy	Treatment Groups after the Policy	Control Groups before the Policy	Control Groups after the Policy
Mean	2.85	3.09	2.74	3.08
**N**	419	560	756	1323

**Table 3:** Impact of LCCP on carbon emission.

	LnCO_2_				
	(1)	(2)	(3)	(4)	(5)
*DID*	−0.029 **	−0.025 *	−0.026 **	−0.024 *	−0.026 **
	(0.01)	(0.01)	(0.01)	(0.01)	(0.01)
lngdp		0.717 ***	0.718 ***	0.607 ***	0.595 ***
		(0.11)	(0.11)	(0.12)	(0.12)
lngdp2		−0.038 ***	−0.038 ***	−0.035 ***	−0.035 ***
		(0.01)	(0.01)	(0.01)	(0.01)
lnpop			0.009 *	0.010 **	0.011 **
			(0.01)	(0.00)	(0.01)
strind				0.217 **	0.220 **
				(0.10)	(0.10)
strpub					0.309
					(0.24)
lnarea					−0.010
					(0.05)
City FE	YES	YES	YES	YES	YES
Time FE	YES	YES	YES	YES	YES
N	3058	3058	3058	3058	3058
R2	0.844	0.864	0.864	0.865	0.865

Standard deviations are in brackets; *, **, ***, representing remarkable at 10%, 5%, or 1% level.

**Table 4:** Parallel trend.

	Pre3	Pre2	Current	Post1	Post2	Post3
	(1)	(2)	(3)	(4)	(5)	(6)
LnCO_2_	−0.007	−0.008	−0.007 **	−0.022 **	−0.022 **	−0.049 ***
	(0.013)	(0.007)	(0.003)	(0.010)	(0.010)	(0.014)
Control variable	YES	YES	YES	YES	YES	YES
City FE	YES	YES	YES	YES	YES	YES
Time FE	YES	YES	YES	YES	YES	YES
N	3058	3058	3058	3058	3058	3058

Standard deviations are in brackets; **, ***, representing remarkable at 5%, 1% level.

**Table 5:** Impact of LCCP on carbon emission (PSM-DID).

	LnCO_2_	
	(1)	(2)
*DID*	−0.031 **	−0.029 **
	(0.013)	(0.012)
Control variable	NO	Yes
City FE	Yes	Yes
Time FE	Yes	Yes
N	3058	3058

Standard deviations are in brackets; **, representing remarkable at 5% level.

**Table 6:** Policy time change.

	LnCO_2_		
	2008	2009	2015
*DID*	−0.022	−0.021	−0.017
	(0.028)	(0.029)	(0.011)
Control Variable	Yes	Yes	Yes
City FE	Yes	Yes	Yes
Time FE	Yes	Yes	Yes
N	3058	3058	3058

Standard deviations are in brackets.

**Table 7:** Sample adjustment.

	LnCO_2_		
	2006–2014	2007–2013	2009–2012
*DID*	−0.025 **	−0.021 *	−0.019 **
	(0.011)	(0.013)	(0.009)
Control Variable	Yes	Yes	Yes
City FE	Yes	Yes	Yes
Time FE	Yes	Yes	Yes
N	2216	1939	1108

Standard deviations are in brackets; *, ** representing remarkable at 10%, 5% level.

**Table 8:** Replace the dependent variable.

	lncogdp	
	(1)	(2)
*DID*	−0.041 ***	−0.026 **
	(0.014)	(0.013)
Control Variables	NO	Yes
City FE	Yes	Yes
Time FE	Yes	Yes
N	3058	3058

Standard deviations are in brackets; **, *** representing remarkable at 5%, 1% level.

**Table 9:** Different estimation methods.

	LnCO_2_			
	FE	RE	MLE	PA
*DID*	−0.026 **	−0.029 **	−0.029 ***	−0.029 **
	(0.013)	(0.013)	(0.006)	(0.013)
Control Variable	Yes	Yes	Yes	Yes
City FE	Yes	Yes	Yes	Yes
Time FE	Yes	Yes	Yes	Yes
N	3058	3058	3058	3058

Standard deviations are in brackets **, *** representing remarkable at 5%, 1% level.

**Table 10:** Eliminate other policy distractions.

	LnCO_2_	
	So2 Emission Trading Pilot	Air Pollution Key Control Area
*DID*	−0.036 *	−0.021 **
	(0.018)	(0.010)
Control Variable	Yes	Yes
City FE	Yes	Yes
Time FE	Yes	Yes
N	1155	3058

Standard deviations are in brackets; *, ** representing remarkable at 10%, 5% level.

**Table 11:** Mechanism testing.

	Innovative Carbon Reduction		Efficient Carbon Reduction		Productive Carbon Reduction			
	(1)	(2)	(3)	(4)	(5)	(6)	(7)	(8)
	R&D	LnCO_2_	Energdp	LnCO_2_	Lnfdi	LnCO_2_	Lnind	LnCO_2_
*DID*	10.635 ***	−0.014	−1.066 *	−0.014	−0.301 ***	−0.020	−0.093 ***	−0.024 *
	(3.707)	(0.013)	(0.577)	(0.011)	(0.085)	(0.013)	(0.025)	(0.013)
R&D		−0.001 ***						
		(0.000)						
Energdp				0.011 ***				
				(0.001)				
Lnfdi						0.002 *		
						(0.001)		
Lnind								0.017
								(0.016)
Control Variable	Yes	Yes	Yes	Yes	Yes	Yes	Yes	Yes
City FE	Yes	Yes	Yes	Yes	Yes	Yes	Yes	Yes
Time FE	Yes	Yes	Yes	Yes	Yes	Yes	Yes	Yes
N	3058	3058	3058	3058	3058	3058	3058	3058

Standard deviations are in brackets; *, *** representing remarkable at 10%, 1% level.

**Table 12:** Heterogeneity of city size.

Unit: 10 Thousand Persons	LnCO_2_			
	Small and Medium-Sized City	Model II Big City	Model I Big City	Super-Large City
	<100	100 ≤& < 300	300 ≤ & < 500	≥500
LnCO_2_	0.127	−0.027	−0.043 **	−0.027 **
	(0.110)	(0.020)	(0.018)	(0.014)
Control Variable	YES	YES	YES	YES
City FE	YES	YES	YES	YES
Time FE	YES	YES	YES	YES
N	130	1027	873	1028

Standard deviations are in brackets; ** representing remarkable at 5% level.

**Table 13:** Heterogeneity of urban characteristics.

	LnCO_2_				
	(1)	(2)	(3)	(4)	(5)
High Digitization	−0.036 ***				
	(0.013)				
Low Digitization	0.001				
	(0.018)				
High Marketization		−0.056 ***			
		(0.013)			
Low Marketization		0.001			
		(0.018)			
High Share of SOEs			0.034 *		
			(0.020)		
Low Share of SOEs			−0.044 ***		
			(0.014)		
High So2				−0.030 **	
				(0.012)	
Low So2				−0.022	
				(0.016)	
HighPM2.5					−0.044 ***
					(0.012)
Low PM2.5					−0.015
					(0.016)
Control Variables	YES	YES	YES	YES	YES
City FE	YES	YES	YES	YES	YES
Time FE	YES	YES	YES	YES	YES

Standard deviations are in brackets; *, **, ***, representing remarkable at 10%, 5%, or 1% level.

**Table 14:** Synergy Effect test.

	LnCO_2_			
	(1)	(2)	(3)	(4)
*DID*	−0.026 **	−0.016	−0.018	−0.037 ***
	(0.013)	(0.015)	(0.013)	(0.012)
*DID* * Digitization		−0.017 *		
		(0.010)		
Digitization		0.000		
		(0.000)		
*DID* * Marketization			−0.023 ***	
			(0.004)	
Marketization			0.005	
			(0.008)	
*DID* * PM2.5				−0.002 ***
				(0.001)
PM2.5				−0.000
				(0.000)
Control Variables	YES	YES	YES	YES
City FE	YES	YES	YES	YES
Time FE	YES	YES	YES	YES
N	3058	3058	3058	3058

Standard deviations are in brackets; *, **, ***, representing remarkable at 10%, 5%, or 1% level.

## Data Availability

The data underlying this article will be shared on reasonable request to the corresponding author (lidandan20221221@163.com).
